# Overt hyperthyroidism is associated with increased dispersion of ventricular repolarization: a case-control study

**DOI:** 10.3389/fendo.2026.1897088

**Published:** 2026-07-07

**Authors:** Maksymilian Kłosowicz, Magdalena Urbańczuk, Aleksandra Burbelka, Agnieszka Gala-Błądzińska, Krzysztof Balawender

**Affiliations:** 1 Department of Normal and Clinical Anatomy, Faculty of Medicine, Medical College, University of Rzeszów, Rzeszów, Poland; 2 Clinical Department of Cardiology with the Acute Coronary Syndromes Subdivision, St. Queen Jadwiga Clinical Hospital in Rzeszow, Rzeszow, Poland; 3 Department of Internal Medicine, Nephrology and Endocrinology, St. Queen Jadwiga Clinical Hospital in Rzeszow, Rzeszow, Poland; 4 Department of Nephrology and Endocrinology, Faculty of Medicine, Medical College, University of Rzeszów, Rzeszów, Poland; 5 Department of Nephrology and Dialysis Unit, Fryderyk Chopin University Clinical Hospital in Rzeszów, Rzeszów, Poland

**Keywords:** electrocardiography, overt hyperthyroidism, repolarization abnormalities, thyroid hormones, ventricular repolarization dispersion

## Abstract

**Background:**

Overt hyperthyroidism is associated with increased cardiovascular morbidity, including tachyarrhythmias and heart failure. Although standard electrocardiographic abnormalities are well recognized, data regarding advanced markers of ventricular repolarization heterogeneity remain limited. We aimed to evaluate electrocardiographic dispersion parameters as markers of altered ventricular repolarization in patients with overt hyperthyroidism.

**Methods:**

This single-centre retrospective case–control study included 97 adults: 55 patients with overt hyperthyroidism and 42 healthy euthyroid controls. Six ventricular repolarization dispersion parameters were assessed from standard 12-lead ECG recordings: QT dispersion (QTd), Bazett-corrected QT dispersion (QTcBd), Fridericia-corrected QT dispersion (QTcFd), JT dispersion (JTd), Fridericia-corrected JT dispersion (JTcFd), and Tpeak–Tend dispersion (Tp-ed). Group comparisons, correlation analyses, receiver operating characteristic (ROC) analyses, and internally validated multivariable modelling were performed.

**Results:**

All six parameters were significantly higher in overt hyperthyroidism than in controls (all p < 0.001). The best discriminatory performance was observed for JTcFd (AUC 0.813) and QTcBd (AUC 0.804). In multivariable analysis, Tp-ed remained independently associated with overt hyperthyroidism. Correlations between thyroid hormone levels and dispersion indices were weak and non-significant.

**Conclusions:**

Our findings suggest an association between overt hyperthyroidism and increased ventricular repolarization heterogeneity, as reflected by alterations in ECG-derived repolarization parameters. These results support the presence of electrophysiological changes in overt hyperthyroidism; however, the clinical implications regarding arrhythmic risk remain uncertain, as no clinical arrhythmic outcomes were assessed.

## 1 Introduction

In countries with adequate iodine intake, hyperthyroidism affects approximately 0.2–2.5% of the population. Overt hyperthyroidism (OHT), defined as low thyroid-stimulating hormone (TSH) levels with elevated free triiodothyronine (FT3) and/or free thyroxine (FT4), occurs in approximately 0.2–1.4% of the population (1). Excess FT3 and FT4 leads to multisystem disturbances, including metabolic changes (weight loss, hyperglycemia, and catabolism resulting in muscle mass loss), neuropsychiatric symptoms such as agitation, insomnia, emotional lability, and irritability, as well as cardiovascular complications, including an increased risk of arrhythmias and the development of heart failure (2–5).

Cardiovascular changes can be readily identified using the rapid and minimally invasive electrocardiographic examination (ECG) (6). Current electrocardiographic markers routinely used in ECG assessment reflect only part of the cardiovascular abnormalities that may be observed in patients with OHT. The advanced electrocardiographic markers proposed in our study, including QT dispersion (QTd), rate-corrected QT dispersion using Bazett’s formula (QTcBd), rate-corrected QT dispersion using Fridericia’s formula (QTcFd), JT dispersion (JTd), rate-corrected JT dispersion using Fridericia’s formula (JTcFd), and T-peak to T-end dispersion (Tp-ed), more accurately characterize ventricular repolarization heterogeneity than conventional ECG parameters.

To date, data regarding the significance of advanced markers of ventricular repolarization in patients with OHT group remain limited. The aim of the present study was to evaluate these parameters and characterize ventricular repolarization abnormalities in patients with OHT.

## 2 Material and methods

### 2.1 Study objectives

The primary objective of this study was to compare ventricular repolarization dispersion parameters between patients with overt hyperthyroidism and healthy euthyroid controls and to assess their association with overt hyperthyroidism. Secondary objectives comprised: (a) characterization and comparison of demographic and clinical profiles between patients with overt hyperthyroidism and healthy euthyroid controls; (b) assessment of the correlation structure between thyroid function markers and dispersion indices within the hyperthyroidism subgroup; and (c) construction of a parsimonious, internally validated multivariable model incorporating propensity-score–based covariate balancing to mitigate confounding.

### 2.2 Study design and structure

**Figure 1:**
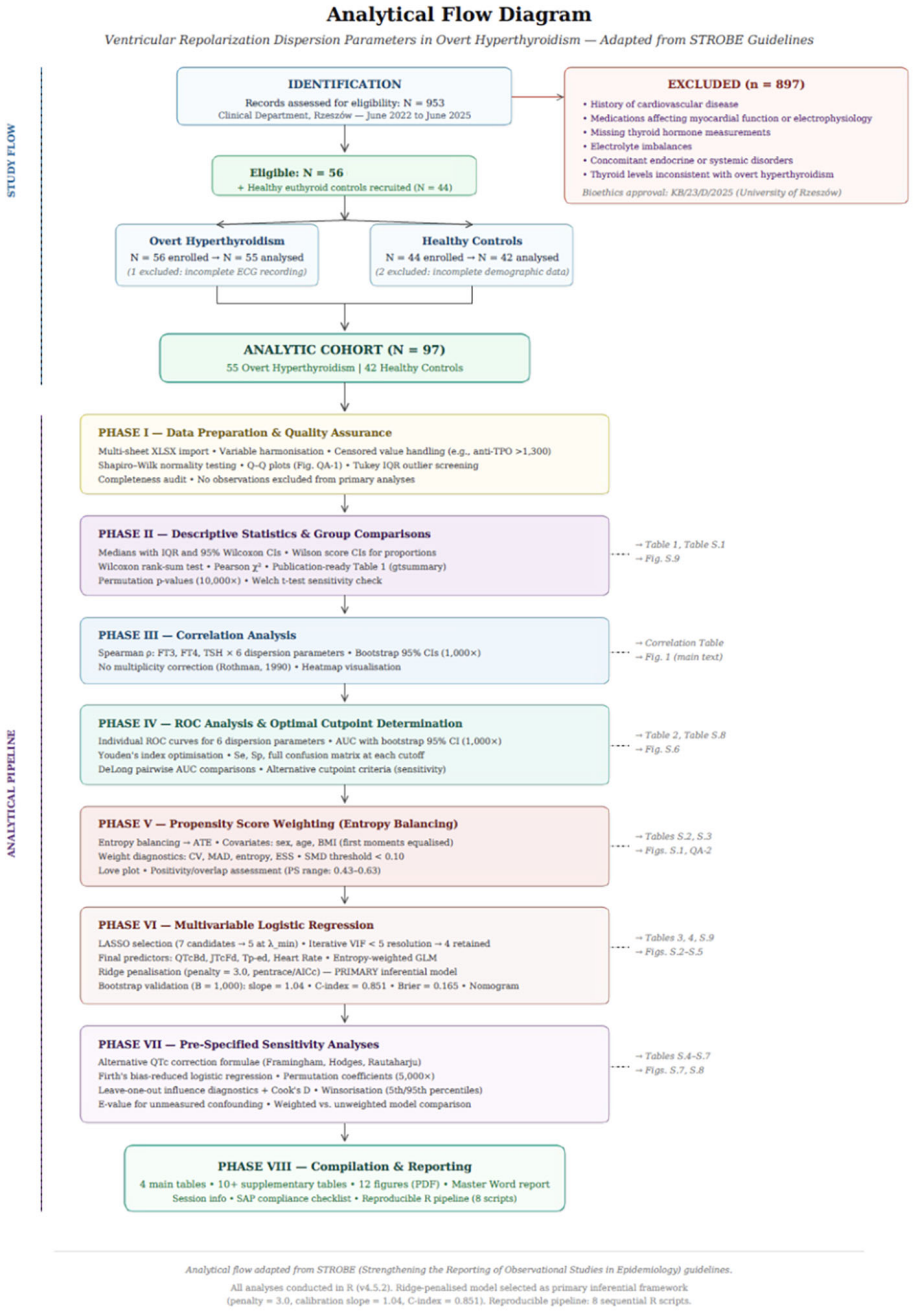
Study flow and analytical architecture for evaluating ventricular repolarization dispersion parameters in overt hyperthyroidism.

This investigation was designed as a single-centre, retrospective, cross-sectional, case–control study. Data were obtained from patients treated at the Clinical Department of Internal Medicine, Nephrology and Endocrinology, including the Nuclear Medicine Unit and Dialysis Center, as well as from the Emergency Department of the Clinical Provincial Hospital named after St. Queen Jadwiga in Rzeszów. The study cohort comprised two groups: patients diagnosed with overt hyperthyroidism (OHT group, N = 56) and healthy euthyroid controls (Control group, N = 44), yielding a total enrolled sample of 100 individuals, of whom 97 constituted the final analytic cohort following exclusion of three participants with incomplete data (**Figure 1**). The study protocol received approval from the Bioethics Committee - approval no. KB/23/D/2025.

Biometric and laboratory data of the enrolled patients were obtained from the hospital’s electronic database. The collected variables were then recorded in a purpose-built Microsoft Excel file created for this study, where they were systematically organized, coded, and prepared for statistical analysis. To ensure confidentiality, all data were anonymized and patients were identified using unique codes instead of personal information. These data were complemented by information from paper medical records as well as available electrocardiographic (ECG) recordings. When more than one ECG was available, the recording closest in time to thyroid hormone measurement was selected for analysis.

As this was a retrospective observational study, the results demonstrate association rather than causation and do not establish predictive relationships.

### 2.3 Inclusion and exclusion criteria

Patients aged ≥18 years hospitalized between June 2022 and June 2025 with a documented diagnosis of hyperthyroidism were included. Exclusion criteria comprised a history of cardiovascular disease, use of medications affecting myocardial function or cardiac electrophysiology, missing thyroid hormone measurements, electrolyte imbalances, concomitant endocrine or systemic disorders influencing hormonal status, and thyroid hormone levels not consistent with OHT.

The complete participant flow – from initial screening of 953 hospital records through application of exclusion criteria to the final analytic cohort of 97 individuals – together with the eight-phase analytical architecture governing all downstream statistical procedures, is depicted in **Figure 1**.

### 2.4 Methodology

#### 2.4.1 Variables and estimands

The primary outcome (dependent variable) was group membership, coded as a binary indicator: OHT (1) versus Control (0), defined by suppressed TSH with concomitantly elevated FT3 and/or FT4 levels. The primary exposure variables (candidate predictors) consisted of the six electrocardiographic dispersion parameters enumerated above, each quantifying the spatial heterogeneity of ventricular repolarization as the difference between maximum and minimum interval values measured across all 12 standard ECG leads. Heart rate (HR, beats per minute) served as an additional candidate predictor owing to its established pathophysiological relevance in hyperthyroidism-associated tachycardia. The target estimand was the average treatment effect in the population (ATE), estimated through entropy-balanced propensity score weighting. Covariates designated for balancing comprised sex (binary), age (continuous, years), and body mass index (BMI, continuous, kg/m^2^). Supplementary laboratory and haematological variables – including anti-TPO, anti-TG, TRAb, lipid panel, glucose, creatinine, estimated glomerular filtration rate, haemoglobin, haematocrit, and electrolytes (sodium, potassium, chloride, calcium)–were collected for descriptive profiling of the OHT subgroup.

#### 2.4.2 Data extraction

All relevant laboratory parameters and additional clinical data were retrieved from the hospital’s electronic medical record system. Electrocardiographic recordings were obtained from the patients’ medical records and subsequently subjected to detailed analysis.

#### 2.4.3 ECG parameters analysis

A standard 12-lead ECG was recorded at a paper speed of 25 mm/s and a calibration of 10 mm/mV, with all measurements performed in patients resting in the supine position. Electrocardiographic parameters were assessed digitally using ImageJ software (National Institutes of Health, Bethesda, MD, USA). Prior to analysis, calibration was verified based on the standard ECG grid: horizontal scaling was confirmed using a 1-second interval (corresponding to five large squares, i.e., 1000 ms), while vertical scaling was referenced to 1 mV, equivalent to 10 mm.

Because several ECG parameters were measured manually, some degree of observer-dependent variability is unavoidable. Although established measurement techniques were used, formal assessment of interobserver and intraobserver reproducibility was beyond the scope of the present study and should be addressed in future investigations.

The definitions and measurement methods of the analyzed electrocardiographic parameters are presented in **Table 1**.

**Table 1:** Overview of definitions, formulas, and measurement techniques for electrocardiographic indices of chosen ventricular repolarization markers.

Parameter	Definition	Formula	Measurement method	Unit
QTd	Difference between the maximum and minimum QT interval across 12 leads	QT max – QT min	QT measured from QRS onset to the end of the T wave in all measurable leads; maximum and minimum values identified across 12 leads	ms
QTcFd	Corrected QT dispersion using Fridericia formula	QTcF max – QTcF min	QT intervals corrected using Fridericia formula (QTcF = QT/RR^(1/3)) in each lead; dispersion calculated from max–min values	ms
QTcBd	Corrected QT dispersion using Bazett formula	QTcB max – QTcB min	QT intervals corrected using Bazett formula (QTcB = QT/√RR) in each lead; dispersion calculated from max–min values	ms
JTd	Difference between the maximum and minimum JT interval across 12 leads	JT max – JT min	JT calculated as QT − QRS in each lead; maximum and minimum JT values identified across all leads	ms
JTcFd	Corrected JT dispersion using Fridericia formula	JTcF max – JTcF min	JT intervals corrected for heart rate using Fridericia formula (JTc = QTc − QRS); dispersion calculated from max–min values	ms
Tp-ed	Difference between the maximum and minimum Tp-e interval across 12 leads	Tp-e max – Tp-e min	Tp-e measured from the peak of the T wave to its end in each lead; dispersion calculated as difference between maximum and minimum values	ms

All electrocardiographic measurements were precisely assigned to the corresponding patients and systematically entered into a dedicated Excel database designed for consistent data organization and subsequent analysis.

**Figure 2:**
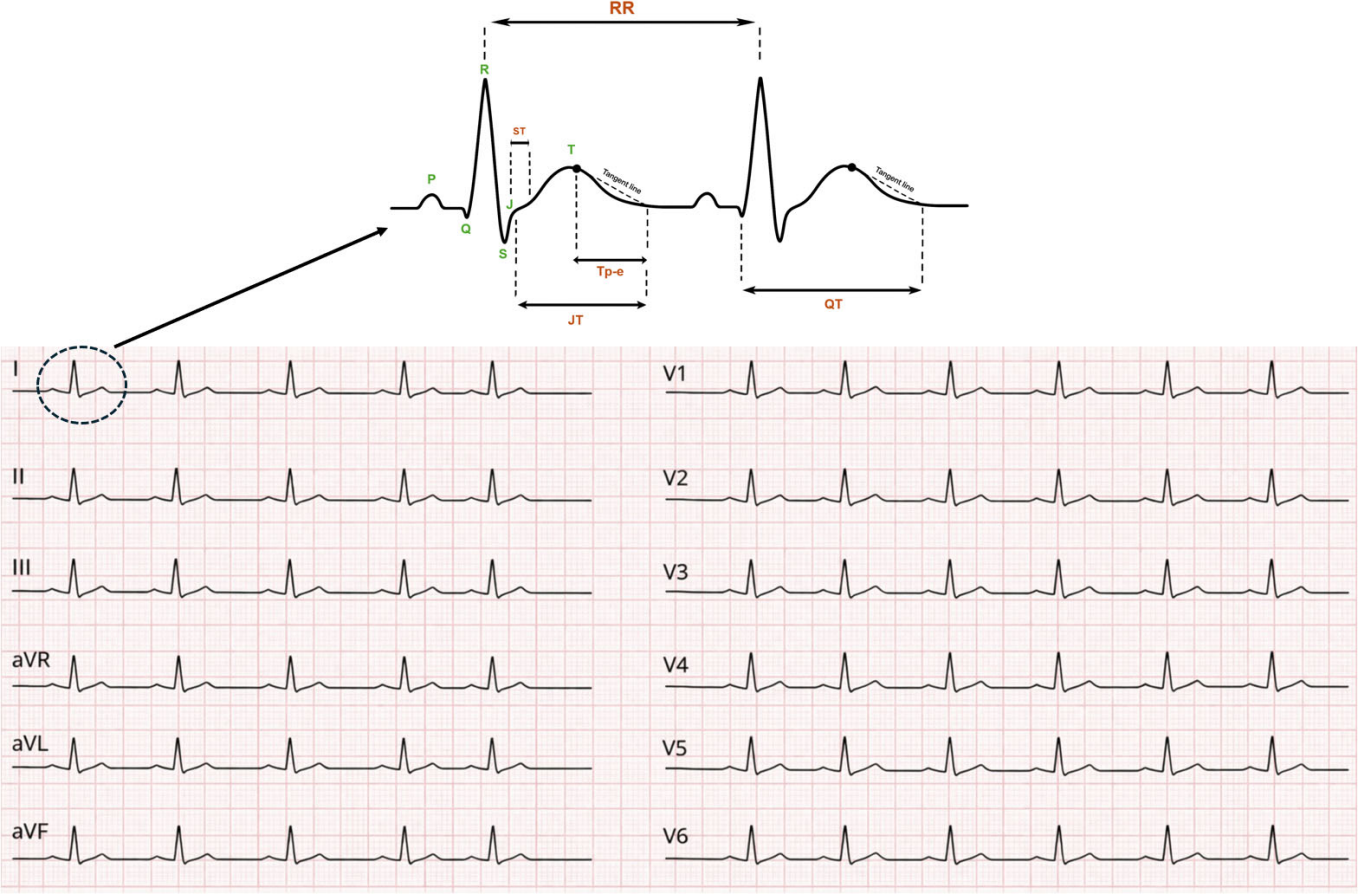
Methodology of electrocardiographic measurements. Measurements were performed in all leads using an identical approach, except for those with negative or indiscernible T waves.

The method for determining the respective ECG intervals is illustrated in **Figure 2**. Detailed measurement procedures are provided in the **Supplementary Materials**.

### 2.5 Statistical analysis and entropy balance

To reduce baseline differences between cases and controls, entropy balancing was applied to achieve covariate balance across age, sex, and body mass index. Before weighting, a mild imbalance was present, whereas after weighting all covariates achieved excellent balance. Importantly, the weighted analyses yielded results that were highly consistent with the unadjusted findings, indicating that the observed associations were not driven by baseline differences between groups. Detailed balance diagnostics, propensity score distributions, and weighting performance are provided in the **Supplementary Materials**.

Detailed descriptions of the statistical methods, sensitivity analyses, software environment, and reproducibility procedures are provided in the **Supplementary Material**.

## 3 Results

### 3.1 Demographic, clinical, and electrocardiographic characteristics

The analytic cohort comprised 97 individuals, of whom 55 had been diagnosed with OHT and 42 served as healthy euthyroid controls. The demographic and clinical characteristics of the two groups are summarised in **Table 2**.

**Table 2:** Demographic, clinical, and electrocardiographic dispersion characteristics of patients with overt hyperthyroidism and healthy controls: comparable baseline profiles with significantly elevated repolarization heterogeneity in the OHT group (N = 97).

Characteristic	OHT (N = 55)	Control (N = 42)	p
I. Demographic characteristics			
Age (years)	46.00 (35.00–59.00), [25.00, 82.00]	46.50 (38.00–59.00), [20.00, 85.00]	.746
Sex			.300
*Female*	43 (78.2%)	28 (66.7%)	
*Male*	12 (21.8%)	14 (33.3%)	
BMI (kg/m^2^)	21.78 (20.52–24.50), [17.00, 29.27]	22.75 (21.20–23.70), [18.50, 26.80]	.675
II. Clinical parameters			
TSH (µIU/mL)	0.01 (0.01–0.01), [0.00, 0.53]	1.74 (1.47–2.41), [0.69, 3.90]	<.001
Heart rate (bpm)	102.00 (93.00–115.00), [65.00, 152.00]	74.44 (65.79–87.72), [47.06, 417.00]	<.001
III. ECG dispersion parameters			
QTd (ms)	37.00 (31.00–46.00), [15.00, 74.00]	26.00 (17.00–35.00), [10.00, 67.00]	<.001
QTcBd – Bazett (ms)	45.85 (38.79–58.30), (7)	30.66 (20.29–39.76), [10.78, 77.78]	<.001
QTcFd – Fridericia (ms)	42.31 (36.39–52.94), [19.03, 91.10]	29.11 (19.11–37.29), [10.51, 74.01]	<.001
JTd (ms)	36.00 (28.00–46.00), [17.00, 81.00]	23.50 (16.00–30.00), [7.00, 84.00]	<.001
JTcFd – Fridericia (ms)	41.05 (34.46–53.97), (8)	25.76 (18.07–32.05), [7.54, 92.79]	<.001
Tp-ed (ms)	29.00 (21.00–40.00), [7.00, 64.00]	21.00 (17.00–28.00), [8.00, 39.00]	<.001

Data are presented as median (Q1–Q3), [min, max] for continuous variables and *n* (%) for categorical variables. *p*-values from the Wilcoxon rank-sum test (continuous) and Pearson’s chi-squared test (categorical). Permutation-based *p*-values (10, 000 resamples) corroborated all results. QTd, QT dispersion; QTcBd, corrected QT dispersion (Bazett); QTcFd, corrected QT dispersion (Fridericia); JTd, JT dispersion; JTcFd, corrected JT dispersion (Fridericia); Tp-ed, Tp-e dispersion; BMI, body mass index; TSH, thyroid-stimulating hormone.

Median age was virtually identical between the OHT and Control groups (46.00 years [IQR 35.00–59.00] vs. 46.50 years [IQR 38.00–59.00]; *p* = .746). The sex distribution favoured females in both groups, consistent with the well-established female predominance of thyroid disease, although the difference did not attain statistical significance (78.2% vs. 66.7% female; *p* = .300). Body mass index was comparable across groups (21.78 vs. 22.75 kg/m^2^; *p* = .675), with both medians falling within the normal range.

Clinically, profound differences emerged that reflect the hypermetabolic state characteristic of OHT. Thyroid-stimulating hormone was markedly suppressed in the OHT group (median 0.01 µIU/mL [IQR 0.01–0.01] vs. 1.74 µIU/mL [IQR 1.47–2.41]; *p* <.001), confirming the diagnostic classification. Resting heart rate was significantly elevated in OHT patients (102.00 bpm [IQR 93.00–115.00]) relative to controls (74.44 bpm [IQR 65.79–87.72]; *p* <.001), a finding consistent with the chronotropic effects of thyroid hormone excess on sinoatrial node automaticity. The biochemical and haematological profile of the OHT subgroup, detailed in **Supplementary Table S1**, revealed profoundly deranged thyroid function (median FT3: 8.52 pg/mL; median FT4: 3.28 ng/dL) alongside preserved renal function (eGFR: 110.50 mL/min/1.73 m^2^), stable electrolyte homeostasis (Na: 139.50, K: 4.20, Cl: 106.00, Ca: 9.40), and mildly reduced haemoglobin (12.90 g/dL), the latter attributable to hypermetabolism-associated haemodilution rather than overt anaemia.

The principal findings of this investigation are evident in the electrocardiographic dispersion parameters. All six indices were significantly elevated in the OHT group compared with controls (all *p* <.001). Uncorrected QT dispersion was increased by approximately 42% (37.00 ms [IQR 31.00–46.00] vs. 26.00 ms [IQR 17.00–35.00]). Rate-corrected QT dispersion amplified this difference further: QTcBd reached 45.85 ms in the OHT group versus 30.66 ms in controls – a relative increase of approximately 50% – while QTcFd exhibited a comparable pattern (42.31 vs. 29.11 ms). The JT-derived dispersions, which isolate the repolarization component by excluding the depolarization phase, demonstrated similarly pronounced elevations: JTd was 53% higher in the OHT group (36.00 vs. 23.50 ms) and JTcFd was 59% higher (41.05 vs. 25.76 ms). Tp-e dispersion, reflecting transmural heterogeneity of repolarization across the ventricular wall, was 38% greater in OHT patients (29.00 vs. 21.00 ms). These observations are visualised in **Supplementary Figure S8**, where violin-box-jitter plots illustrate not only the significant between-group separation in central tendency but also the partial distributional overlap, indicating that while the group-level effect is robust, individual-level classification requires multivariable approaches explored in subsequent sections.

The consistency of these findings across all six parameters – spanning three distinct families of repolarization metrics (QT-derived, JT-derived, and Tp-e–derived) – reinforces the interpretation that overt hyperthyroidism is associated with a global increase in the spatial heterogeneity of ventricular repolarization, rather than an isolated perturbation of any single electrophysiological domain. The magnitude of elevation was greatest for the rate-corrected JT-family indices, an observation that carries particular pathophysiological weight given that JT-based parameters are independent of depolarization duration and thus may more faithfully capture pure repolarization abnormalities, especially in the tachycardic setting characteristic of this population. Permutation-based *p*-values (10, 000 resamples) corroborated the asymptotic Wilcoxon results for all six parameters, confirming that the observed significances are not artefacts of distributional violations inherent to the modest sample size. Visual assessment of distributional properties (**Supplementary Figure QA-1**) corroborated these findings.

### 3.2 Correlation between thyroid function markers and dispersion parameters

**Figure 3:**
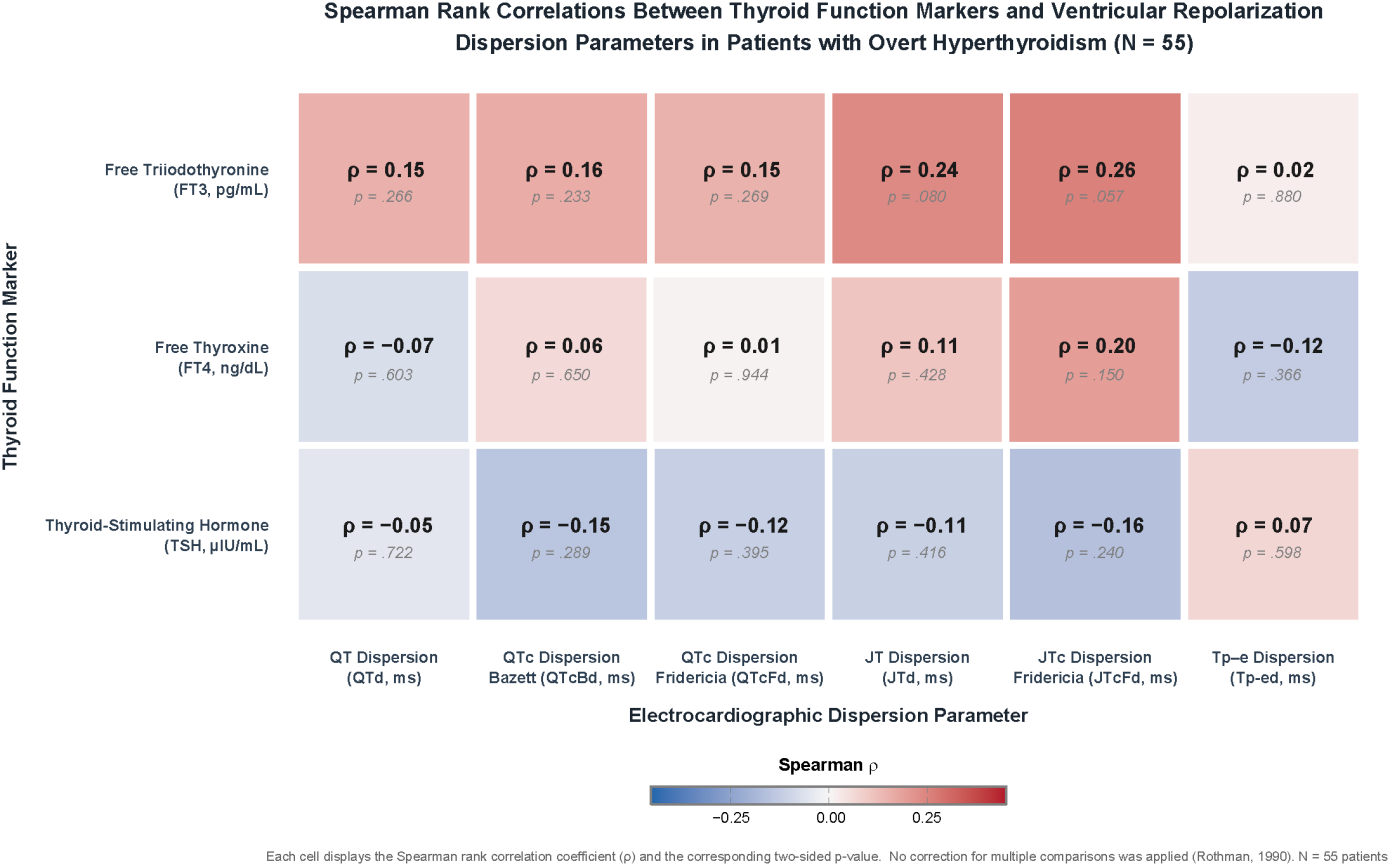
Spearman rank correlations between thyroid function markers and ventricular repolarization dispersion parameters reveal limited monotonic associations within the hyperthyroidism subgroup (N = 55). Each cell displays the Spearman rank correlation coefficient (ρ) and the corresponding two-sided p-value. Colour intensity encodes the magnitude and direction of the association (red = positive, blue = negative). No correction for multiple comparisons was applied (Rothman, 1990).

Within the OHT subgroup (N = 55), Spearman rank correlations were computed between three thyroid function markers – free triiodothyronine (FT3), free thyroxine (FT4), and thyroid-stimulating hormone (TSH) – and the six electrocardiographic dispersion parameters. The heatmap in **Figure 3** provides a comprehensive visual summary of all pairwise associations, facilitating rapid identification of correlation patterns. The complete matrix of correlation coefficients with bootstrap 95% confidence intervals is reported in **Supplementary Table S10**, which confirms that no single pairwise association yielded a confidence interval excluding zero.

The overwhelming majority of correlations were weak in magnitude and statistically non-significant, indicating that within this subgroup – all of whom exhibited biochemically confirmed OHT – the degree of thyroid hormone elevation bore limited monotonic relationship to the spatial heterogeneity of ventricular repolarization.

The strongest associations were observed between FT3 and the JT-derived dispersion indices: FT3 × JTcFd (ρ = 0.258, 95% CI [−0.004, 0.480], p = .057) and FT3 × JTd (ρ = 0.238, 95% CI [−0.015, 0.450], p = .080). Although neither reached conventional significance, both exhibited confidence intervals whose lower bounds were only marginally below zero. The directionality – positive, indicating that higher circulating FT3 levels tend to accompany greater JT-based repolarization heterogeneity – is pathophysiologically coherent, given the established effects of triiodothyronine on potassium and calcium channel expression in cardiomyocytes. The preferential coupling of FT3 (rather than FT4) with dispersion indices aligns with the recognition that FT3 constitutes the biologically active thyroid hormone at the myocardial level. In contrast, FT4 demonstrated negligible correlations across all dispersion parameters (ρ ranging from −0.124 to 0.197, all p >.15), as did TSH (ρ ranging from −0.161 to 0.071, all p >.24). The absence of meaningful TSH associations is expected given the profoundly suppressed and uniformly near-zero TSH values within this subgroup (median 0.01 µIU/mL), which afford minimal inter-individual variability for correlation analysis.

These findings carry two implications. First, the between-group differences in dispersion parameters documented in the preceding section are attributable to the categorical presence versus absence of OHT rather than to a graded, dose–response relationship with hormone concentration within the hyperthyroid state. Second, the near-significant FT3–JTcFd association warrants investigation in larger cohorts with broader hormonal ranges, where adequate statistical power may reveal a genuine, albeit modest, monotonic dependency obscured by the restricted dynamic range of the present sample.

### 3.3 Discriminatory performance of dispersion parameters

Receiver operating characteristic (ROC) analysis was performed for each of the six dispersion parameters individually, with discriminatory performance metrics summarised in **Table 3** and individual ROC curves with optimal operating points depicted in **Supplementary Figure S6**.

**Table 3:** Receiver operating characteristic analysis of ECG dispersion parameters for differentiating patients with overt hyperthyroidism from healthy controls within the study cohort.

Parameter	n	Cut-off	J	Acc.	Se	Sp	TP	FN	FP	TN	AUC [95% CI]
QTd (ms)	97	≥ 29.0	0.43	0.71	0.75	0.69	41	14	13	29	0.727 [0.627–0.827]
QTcBd (ms)	97	≥ 37.9	0.50	0.74	0.73	0.76	40	15	10	32	0.804 [0.714–0.895]
QTcFd (ms)	97	≥ 32.7	0.46	0.71	0.64	0.83	35	20	7	35	0.781 [0.685–0.876]
JTd (ms)	97	≥ 28.0	0.48	0.73	0.69	0.79	38	17	9	33	0.765 [0.667–0.862]
JTcFd (ms)	97	≥ 32.7	0.52	0.75	0.71	0.81	39	16	8	34	0.813 [0.721–0.904]
Tp-ed (ms)	97	≥ 29.0	0.40	0.68	0.60	0.81	33	22	8	34	0.725 [0.621–0.829]

Optimal cut-offs determined by maximising Youden’s index (J = Sensitivity + Specificity – 1). TP, true positive; FN, false negative; FP, false positive; TN, true negative; Positive class, overt hyperthyroidism; AUC, area under the ROC curve; 95% CI via 1, 000 stratified bootstrap resamples; Se, sensitivity; Sp, specificity; Acc., accuracy. Pairwise AUC comparisons (DeLong’s test) in **Supplementary Table S8**.

ROC analyses were performed to evaluate the ability of individual ECG dispersion parameters to discriminate between patients with overt hyperthyroidism and healthy controls rather than to assess their utility as standalone diagnostic tools for hyperthyroidism.

Among the six indices evaluated, JTcFd exhibited the highest discriminatory capacity, attaining an AUC of 0.813 (95% CI [0.721–0.904]). At its Youden-optimised cutoff of ≥ 32.7 ms, this parameter achieved a sensitivity of 71%, specificity of 81%, and overall accuracy of 75%, with the highest Youden index in the panel (J = 0.52). QTcBd ranked second, with an AUC of 0.804 (95% CI [0.714–0.895]) and a cutoff of ≥ 37.9 ms yielding balanced sensitivity (73%) and specificity (76%). These two rate-corrected parameters – one from the JT family, the other from the QT family – consistently outperformed their uncorrected counterparts, an observation formally confirmed by pairwise DeLong comparisons (**Supplementary Table S8**), which revealed that both QTcBd and QTcFd discriminated significantly better than uncorrected QTd (Z = −4.28, p <.001 and Z = −4.34, p <.001, respectively), and that JTcFd significantly outperformed JTd (Z = −4.07, p <.001).

QTcFd (AUC = 0.781) and JTd (AUC = 0.765) occupied an intermediate tier, both demonstrating acceptable discrimination with confidence intervals comfortably above the 0.50 chance level. Notably, QTcFd achieved the highest specificity in the panel (83%) at the expense of lower sensitivity (64%). Uncorrected QTd and Tp-ed exhibited the lowest AUC values (0.727 and 0.725, respectively), though neither confidence interval included 0.50, confirming that even the weakest-performing dispersion parameter retained statistically meaningful discriminatory capacity.

The identified ROC-derived thresholds may serve as exploratory reference values within the present study cohort. In particular, JTcFd values above 32.7 ms and QTcBd values above 37.9 ms demonstrated the most favorable balance between sensitivity and specificity for differentiating patients with overt hyperthyroidism from healthy controls in this dataset. However, these cutoffs should be considered exploratory and hypothesis-generating, as external validation in independent cohorts is required before any clinical application can be considered.

The sensitivity analysis employing alternative rate-correction formulae (**Supplementary Table S4**) revealed that Framingham, Hodges, and Rautaharju methods yielded identical AUC values (0.732) when applied to the detailed QT analysis sheet, outperforming Bazett (0.510) and Fridericia (0.509) in that recomputed context. This discrepancy – in which the same correction formulae that performed well on the main analytic dataset showed attenuated performance on the detailed sheet – reflects differences in the source data structure (independently recomputed max/min QT per formula versus pre-calculated dispersions) and warrants interpretive caution regarding the generalisability of correction-method rankings across measurement protocols.

Collectively, these results suggest that rate-corrected dispersion indices – particularly JTcFd and QTcBd – differentiate patients with overt hyperthyroidism from controls within this study population, with AUC values of 0.80–0.81 indicating good discriminatory performance. The identified Youden-optimised cutoffs (JTcFd ≥ 32.7 ms; QTcBd ≥ 37.9 ms) should be considered exploratory and require validation in independent prospective cohorts before any clinical application can be considered.

### 3.4 Multivariable predictive analysis

In weighted multivariable analysis, Tp-e dispersion remained independently associated with OHT, and the internally validated penalised model showed good discrimination and calibration (**Supplementary Results**).

## 4 Discussion

In the present study, we demonstrated that patients with OHT exhibit significant alterations in ventricular repolarization parameters, reflected by increased values of several electrocardiographic parameters. Collectively, these findings are in agreement with the established role of thyroid hormones in modulating cardiac electrophysiology, providing further evidence that endocrine disturbances can be reflected in surface ECG repolarization markers (9). Our results expand current knowledge by providing a comprehensive assessment of novel repolarization markers in this population, highlighting the potential importance of parameters beyond standard analysis.

The observed changes may result from the effects of thyroid hormones on cardiomyocyte ion channels, including potassium currents (IKr, IKs), sodium currents, and calcium currents, leading to alterations in action potential duration. A heterogeneous response of the different myocardial layers, together with increased sympathetic activity, may further enhance repolarization heterogeneity, thereby promoting arrhythmogenesis (9–13).

In another study conducted by our team, we demonstrated that patients with hyperthyroidism exhibit not only increased heterogeneity of ventricular repolarization, but also prolongation of its global duration. This suggests that excess thyroid hormones affect both the spatial dispersion and temporal dynamics of repolarization, which may lead to increased electrical instability of the myocardium (14).

**Figure 4:**
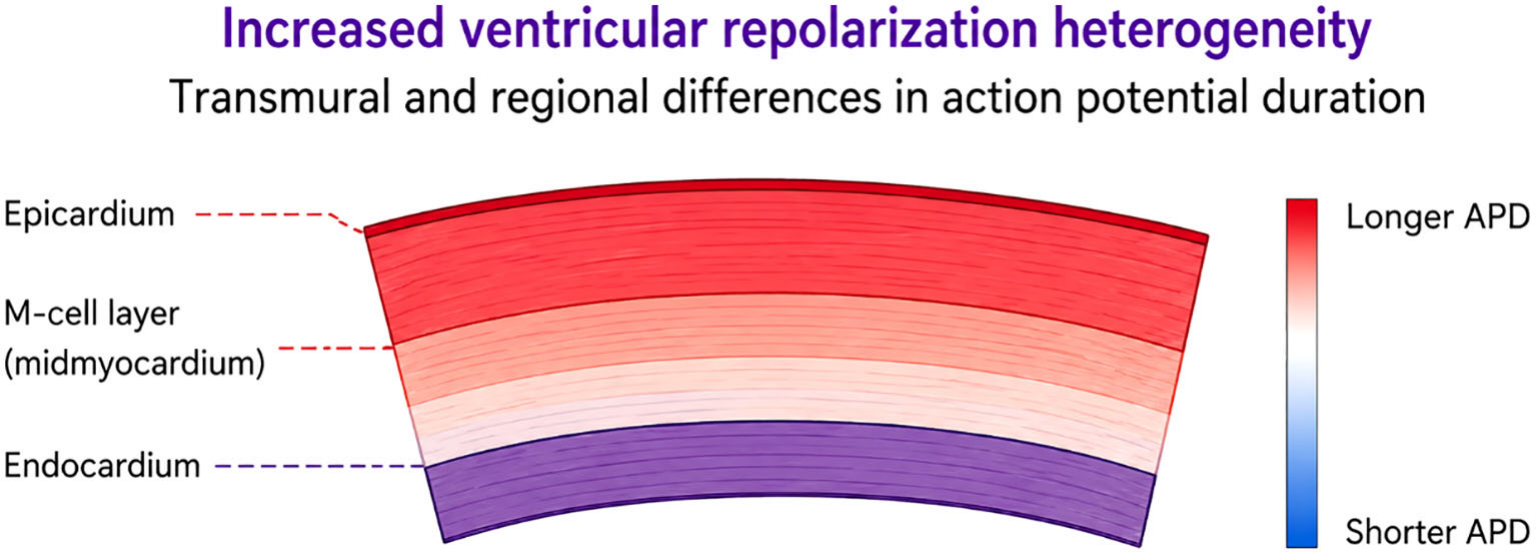
Schematic representation of increased ventricular repolarization heterogeneity in overt hyperthyroidism. Differences in action potential duration (APD) across the ventricular wall layers—epicardium, midmyocardial M-cell layer, and endocardium—illustrate enhanced transmural and regional dispersion of repolarization. Red shading denotes longer APD, whereas blue shading indicates shorter APD. Such non-uniform repolarization may contribute to myocardial electrical instability and electrophysiological alterations.

Our study demonstrated significantly greater QT, QTc dispersion, JT, JTc and Tp-e dispersion in patients with overt hyperthyroidism compared with controls, indicating increased spatial heterogeneity of ventricular repolarization. These findings suggest that excess thyroid hormones contribute to non-uniform recovery of ventricular excitability, likely reflecting regional differences in action potential duration across myocardial layers (**Figure 4**). Notably, Tp-e based parameters may provide a more direct assessment of repolarization heterogeneity than conventional QT-derived indices, as they are thought to better capture transmural dispersion of repolarization, a key electrophysiological substrate associated with ventricular electrical instability. Similarly, the observed increase in JT- and JTc-based dispersion measures highlights the potential value of repolarization assessment independent of ventricular depolarization duration. Consequently, increased JT dispersion may represent a more sensitive marker of repolarization abnormalities than conventional QT-based parameters, particularly in the presence of tachycardia, a characteristic feature of overt hyperthyroidism.

Previous studies have shown that elevated QTd values were associated with a higher risk of ventricular arrhythmias in patients with myocardial infarction (15, 16). A positive correlation has also been demonstrated between QTd and QTcd and the extent of coronary artery disease (15, 17, 18). In a study conducted by Elibet Chávez-González et al., increased QTd in patients with acute ST-segment elevation myocardial infarction (STEMI) was identified as a marker of myocardial electrical instability and was associated with the occurrence of life-threatening ventricular arrhythmias, including ventricular tachycardia (VT) and ventricular fibrillation (VF) (19).

Moreover previous studies have shown that QT dispersion increases in the acute phase of stroke, likely reflecting transient autonomic imbalance - particularly enhanced sympathetic activity with a possible additional contribution of lesion localization at later stages (20). Additionally in patients with dilated cardiomyopathy, increase of QTd can be associated with ventricular arrhythmias and can suggest potential value in risk stratification (14, 21).

Currently, only a limited number of studies have focused on JT interval dispersion (JTd) in adult patients for the assessment of arrhythmic risk, whereas most of the available data originate from pediatric populations, which precluded direct comparison of our findings with those reported by other authors.

In a study conducted by Hiroki Shimizu et al., JTd and QTd were compared between patients with polymorphic ventricular tachycardia (PMVT), monomorphic ventricular tachycardia (MVT), and a control group of healthy individuals. JT dispersion was highest in the PMVT/VF group and reflected repolarization abnormalities more accurately than QT dispersion, particularly in the presence of prolonged QRS duration, suggesting its greater utility in arrhythmic risk assessment. These findings indicate that JT-based parameters, being independent of depolarization duration, may better reflect true repolarization abnormalities (22). Changes in JTd and JTcd indicate that excess thyroid hormones affect not only the global duration of repolarization, but primarily its spatial heterogeneity. This may result from their effects on potassium and calcium currents, leading to regional differences in action potential duration across the myocardium (23).

In a study conducted by Castro Hevia J et al. in patients with Brugada syndrome, increased Tp-e dispersion parameters were associated with a higher risk of serious rhythm disturbances, such as ventricular tachycardia and ventricular fibrillation. Tp-ed abnormalities were particularly pronounced in patients with recurrent arrhythmias, whereas they were not significant in individuals without arrhythmic events, supporting their potential usefulness in risk stratification (24).

Similarly, in studies involving patients with ST-segment elevation myocardial infarction (STEMI), Tp-ed values were shown to be increased, particularly in leads corresponding to ischemic regions, and were correlated with the occurrence of serious arrhythmic events (25).

Interestingly, the electrophysiological abnormalities observed in hyperthyroidism may be at least partially reversible. Previous studies have shown that successful treatment of hyperthyroidism leads to normalization of heart rate, regression of hyperdynamic circulatory changes, and improvement of several electrocardiographic abnormalities (26, 27).

An additional consideration is the substantially higher resting heart rate observed in the hyperthyroid group. Although several repolarization indices were corrected for heart rate and heart rate itself was included in the multivariable modelling process, residual confounding cannot be entirely excluded. Given the well-established influence of heart rate on ventricular repolarization measurements, some of the observed differences may partly reflect the physiological consequences of sinus tachycardia accompanying thyroid hormone excess. Future studies with larger cohorts should further explore the independent contributions of heart rate and thyroid hormone status to repolarization heterogeneity.

The obtained results provide additional insight into ventricular repolarization abnormalities in patients with overt hyperthyroidism. The results suggest that standard ECG analysis in patients with hyperthyroidism may not fully capture the complexity of ventricular repolarization abnormalities in this population.

Although the present study was not designed to evaluate arrhythmic risk, the observed alterations support the presence of measurable electrophysiological changes in patients with overt hyperthyroidism.

## 5 Conclusions and future directions

This study demonstrates that OHT is accompanied by a global and substantial increase in the spatial heterogeneity of ventricular repolarization, manifested consistently across all six electrocardiographic dispersion parameters examined (38–59% elevations, all *p* <.001). The magnitude and consistency of these elevations spanning QT-derived, JT-derived, and transmural Tp-e indices indicate that overt hyperthyroidism is associated with broad changes in ventricular repolarization, extending beyond its established effects on heart rate and cardiac automaticity. Collectively, these findings suggest that advanced ECG dispersion markers may provide complementary information to standard ECG assessment in the evaluation of electrophysiological alterations associated with thyroid hormone excess.

Among the six parameters evaluated, rate-corrected JT dispersion (Fridericia) and rate-corrected QT dispersion (Bazett) exhibited the strongest individual discriminatory performance (AUC = 0.813 and 0.804, respectively), with Youden-optimised cutoffs of ≥ 32.7 ms and ≥ 37.9 ms demonstrating balanced sensitivity and specificity in the 71–81% range. The superiority of rate-corrected over uncorrected indices is noteworthy, particularly in a population where resting tachycardia is common. Because heart rate substantially influences ventricular repolarization measurements, uncorrected dispersion values may not fully reflect repolarization heterogeneity in patients with overt hyperthyroidism.

The multivariable model combining QTcBd, JTcFd, Tp-ed, and heart rate captured complementary electrophysiological information that no single parameter could provide in isolation. Tp-e dispersion—reflecting transmural heterogeneity across the ventricular wall—remained independently associated with overt hyperthyroidism. This finding may suggest that alterations in transmural repolarization represent one component of the broader electrophysiological changes observed in overt hyperthyroidism. Notably, within the hyperthyroid subgroup, the severity of hormone elevation bore no meaningful monotonic relationship to dispersion magnitude (strongest correlation: FT3 × JTcFd, ρ = 0.258, *p* = .057). This finding suggests that the presence of overt hyperthyroidism may be more closely associated with alterations in ventricular repolarization than the absolute degree of thyroid hormone elevation. However, these observations should be interpreted cautiously given the limited sample size and restricted hormonal range within the study cohort.

These findings position ventricular repolarization dispersion parameters as accessible, non-invasive markers derivable from routine 12-lead ECG recordings that may provide complementary information regarding ventricular repolarization abnormalities. The identified cutoff values and the internally validated multivariable model (C-index = 0.851, calibration slope = 1.04) require external validation in independent, multicentre cohorts to determine their reproducibility, calibration, and generalisability.

The use of extended parameter, may allow a more precise assessment of repolarization heterogeneity, which represents an important component of myocardial electrical instability and their potential role in routine ECG assessment requires further investigation in prospective studies.

### 5.1 Limitations

This study has several limitations. First, although advanced statistical techniques, including entropy balancing, penalised regression, and bootstrap internal validation, were employed to improve model stability and reduce bias, the study was conducted in a relatively small cohort. Consequently, the reported model performance and identified predictor effects may still be susceptible to residual overfitting despite the use of regularisation and optimism correction. Second, all predictive analyses were developed and validated within the same dataset, and no external validation cohort was available. Therefore, the proposed cut-off values and predictive models should be considered exploratory and hypothesis-generating rather than ready for clinical implementation. Independent prospective studies with larger sample sizes are required to confirm the reproducibility, calibration, and generalisability of these findings before their potential application in routine clinical practice. The study was not designed to evaluate clinical arrhythmic outcomes, therefore no conclusions regarding arrhythmia risk can be drawn. Additionally, ECG parameters were derived from a single recording and partly assessed manually, which may introduce measurement variability.

## Data Availability

The original contributions presented in the study are included in the article/**Supplementary Material**. Further inquiries can be directed to the corresponding author.

## References

[1] Lee SY Pearce EN . Hyperthyroidism: a review. JAMA. (2023) 330:1472–83. doi: 10.1001/jama.2023.19052 PMC10873132 37847271

[2] Chaker L Cooper DS Walsh JP Peeters RP . Hyperthyroidism. Lancet. (2024) 403:768–80. doi: 10.1016/s0140-6736(23)02016-0 38278171

[3] Fan SWD Ong LT . Prevalence and risk factors of heart failure in patients diagnosed with hyperthyroidism: a systematic review and meta-analysis. touchREV Endocrinol. (2024) 20:91–9. doi: 10.17925/ee.2024.20.2.12 39526051 PMC11548352

[4] Wiersinga WM Poppe KG Effraimidis G . Hyperthyroidism: aetiology, pathogenesis, diagnosis, management, complications, and prognosis. Lancet Diabetes Endocrinol. (2023) 11:282–98. doi: 10.1016/s2213-8587(23)00005-0 36848916

[5] Navarro-Navajas A Cruz JD Ariza-Ordoñez N Giral H Palmezano J Bolívar-Mejía A . Cardiac manifestations in hyperthyroidism. Rev Cardiovasc Med. (2022) 23:136. doi: 10.31083/j.rcm2304136 39076244 PMC11273775

[6] Bruce K Moore AK Mello I Hilliard T Day M . Basics of the 12-lead ECG. Nursing. (2023) 53:20–5. doi: 10.1097/01.nurse.0000991560.56637.b6 37856294

[7] Akdi A Tekin Tak B Özcan Çetin EH Çetin MS Yayla Ç . Electrocardiography clues in assessment of patients with premature ventricular contractions. Turk J Med Sci. (2021) 51:2986–93. doi: 10.3906/sag-2012-70 34493030 PMC10734884

[8] Mersha BH Abdissa SG Alemneh TA Kebede N Tsega Y Nigussie S . Magnitude of cardiac abnormality and its associated factors among hyperthyroidism patients on follow-up at Tikur Anbessa Specialized Hospital, Addis Ababa, Ethiopia. BMC Cardiovasc Disord. (2024) 24:558. doi: 10.1186/s12872-024-04212-3 39402443 PMC11475159

[9] Krijger Juárez C Amin AS Offerhaus JA Bezzina CR Boukens BJ . Cardiac repolarization in health and disease. JACC Clin Electrophysiol. (2023) 9:124–38. doi: 10.1016/j.jacep.2022.09.017 36697193

[10] Grais IM Sowers JR . Thyroid and the heart. Am J Med. (2014) 127:691–8. doi: 10.1016/j.amjmed.2014.03.009 24662620 PMC4318631

[11] Kahaly GJ Dillmann WH . Thyroid hormone action in the heart. Endocr Rev. (2005) 26:704–28. doi: 10.1210/er.2003-0033 15632316

[12] Mengistie BT Shakir YF Bekele FD Abera BF Mengistie CT Teferi MG . Atrial fibrillation and associated cardiovascular disorders in adults with hyperthyroidism: a retrospective cohort study from two teaching hospitals. Thyroid Res. (2026) 19. doi: 10.1186/s13044-026-00295-6 42050715 PMC13127038

[13] Alwan H Hysaj O Gencer B Duntas L Rodondi N . Thyroid dysfunction and cardiovascular disease. Eur Heart J. (2026) 47(31):2606–23. doi: 10.1093/eurheartj/ehag248 41996368 PMC13225879

[14] Kłosowicz M Urbańczuk M Burbelka A Gala-Błądzińska A Balawender K . Impact of thyroid hormone imbalance on electrocardiographic parameters: systematic review and meta-analysis. J Clin Med. (2025) 14. doi: 10.3390/jcm14248755 PMC12733553 41464657

[15] Abu Shajahan M Mohideen B P A J Thaha SM Ashraf AR Nazar I . Prognostic value of QTc dispersion in acute myocardial infarction. Cureus. (2025) 17:e82846. doi: 10.7759/cureus.82846 40416232 PMC12102647

[16] Mulay DV Quadri SM . QT dispersion and early arrhythmic risk in acute myocardial infarction. Indian Heart J. (2004) 56:636–41. 15751519

[17] Salari F Nough H Seyedhosseini SM Namayandeh SM . Electrocardiographic markers of arrhythmogenic risk in patients with isolated coronary artery ectasia. Int J Cardiol Cardiovasc Risk Prev. (2025) 27:200492. doi: 10.1016/j.ijcrp.2025.200492 40893921 PMC12392676

[18] Yilmaz R Demirbag R Gur M . The association of QT dispersion and QT dispersion ratio with extent and severity of coronary artery disease. Ann Noninvasive Electrocardiol. (2006) 11:43–51. doi: 10.1111/j.1542-474x.2006.00081.x 16472282 PMC6932384

[19] Chávez-González E Rodríguez-Jiménez AE Ferrer-Rodríguez CJ Donoiu I . Ventricular arrhythmias are associated with increased QT interval and QRS dispersion in patients with ST-elevation myocardial infarction. Rev Port Cardiol. (2022) 41:395–404. doi: 10.1016/j.repc.2021.03.015 36062639

[20] Afsar N Fak AS Metzger JT Van Melle G Kappenberger L Bogousslavsky J . Acute stroke increases QT dispersion in patients without known cardiac diseases. Arch Neurol. (2003) 60:346–50. doi: 10.1001/archneur.60.3.346 12633145

[21] DE Maria E Curnis A Garyfallidis P Mascioli G Santangelo L Calabrò R . QT dispersion on ECG Holter monitoring and risk of ventricular arrhythmias in patients with dilated cardiomyopathy. Heart Int. (2006) 2:33. doi: 10.1177/182618680600200106 21977249 PMC3184657

[22] Shimizu H Ohnishi Y Inoue T Yokoyama M . QT and JT dispersion in patients with monomorphic or polymorphic ventricular tachycardia/ventricular fibrillation. J Electrocardiol. (2001) 34:119–25. doi: 10.1054/jelc.2001.23361 11320459

[23] Klein I Danzi S . Thyroid disease and the heart. Circulation. (2007) 116:1725–35. doi: 10.1161/circulationaha.106.678326 17923583

[24] Castro Hevia J Antzelevitch C Tornés Bárzaga F Dorantes Sánchez M Dorticós Balea F Zayas Molina R . Tpeak-Tend and Tpeak-Tend dispersion as risk factors for ventricular tachycardia/ventricular fibrillation in patients with the Brugada syndrome. J Am Coll Cardiol. (2006) 47:1828–34. doi: 10.1016/j.jacc.2005.12.049 16682308 PMC1474075

[25] Xianpei W Sha W Chuanyu G Juanjuan Y Chong C Yongen S . Tpeak-Tend dispersion as a predictor for malignant arrhythmia events in patients with vasospastic angina. Int J Cardiol. (2017) 249:61–5. doi: 10.1016/j.ijcard.2017.07.093 29121758

[26] Cai Z Dai M Zhang Y Zhong H Tan T Bao M . Imbalance of cardiac autonomic nervous activity and increase of ventricular repolarization dynamicity induced by thyroid hormones in hyperthyroidism. Auton Neurosci. (2018) 213:86–91. doi: 10.1016/j.autneu.2018.06.006 30005745

[27] Nowak J Dubiel J . Influence of hyperthyroidism defined as TSH serum level on QT corrected interval dispersion (QTcd). Przeglad Lekarski. (2006) 63(3):123–7. 16967699

